# Calreticulin expression in human cardiac myocytes induces ER stress‐associated apoptosis

**DOI:** 10.14814/phy2.14400

**Published:** 2020-04-23

**Authors:** Michael W. Stoner, Charles F. McTiernan, Iain Scott, Janet R. Manning

**Affiliations:** ^1^ Division of Cardiology Department of Medicine University of Pittsburgh Pittsburgh PA USA; ^2^ Department of Medicine Vascular Medicine Institute University of Pittsburgh Pittsburgh PA USA; ^3^ Department of Medicine Center for Metabolism and Mitochondrial Medicine University of Pittsburgh PA USA

**Keywords:** AC16 cells, calreticulin, ER stress, heart

## Abstract

The global burden of heart failure following myocardial ischemia‐reperfusion (IR) injury is a growing problem. One pathway that is key to understanding the progression of myocardial infarction and IR injury is the endoplasmic reticulum (ER) stress pathway, which contributes to apoptosis signaling and tissue death. The role of calreticulin in the progression of ER stress remains controversial. We hypothesized that calreticulin induction drives proapoptotic signaling in response to ER stress. We find here that calreticulin is upregulated in human ischemic heart failure cardiac tissue, as well as simulated hypoxia and reoxygenation (H/R) and thapsigargin‐mediated ER stress. To test the impact of direct modulation of calreticulin expression on ER stress‐induced apoptosis, human cardiac‐derived AC16 cells with stable overexpression or silencing of calreticulin were subjected to thapsigargin treatment, and markers of apoptosis were evaluated. It was found that overexpression of calreticulin promotes apoptosis, while a partial knockdown protects against the expression of caspase 12, CHOP, and reduces thapsigargin‐driven TUNEL staining. These data shed light on the role that calreticulin plays in apoptosis signaling during ER stress in cardiac cells.

## Graphical abstract

Calreticulin is induced in cardiac tissue in response to ischemia or ER stress. We found that upregulation of calreticulin induces apoptotic signaling in response to ER stress, and that reduction of calreticulin expression protects against apoptosis.

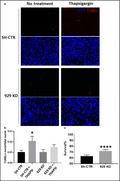

## 1 INTRODUCTION

Endoplasmic reticulum (ER) stress signaling within cardiomyocytes has emerged in recent years as a significant contributor to the progression of cardiac disease. Myocardial ER stress is associated with several cardiovascular diseases, including hypertension, fibrosis, and myocardial infarction (Hong, Kim, Kim, & Park, 2017; Mesaeli, Nakamura, Opas, & Michalak, 2001; Toth et al., 2007). In the context of myocardial infarction and ischemic heart disease, the reported impact of the ER stress response has been mixed, with some groups reporting that ER stress signaling is adaptive and protects the heart from disease progression, and others reporting that ER stress signaling contributes to apoptosis and disease etiology. A resolution of this paradox is necessary for a better understanding of the importance of ER stress in cardiac disease.

The ER stress response is triggered by protein folding defects in the ER. Unfolded or misfolded proteins preferentially bind to the chaperone protein BiP, and as their levels rise, they compete with the upstream effectors that are constitutively bound to BiP under normal conditions (Wang, Xu, Gillette, Jiang, & Wang, 2018). These effectors activate three distinct arms of the ER stress response, comprised of PERK, IRE‐1, and ATF6. Each arm acts to relieve the accumulation of potentially harmful protein aggregates by reducing protein transcription and translation, degrading excess unfolded proteins, or, in extreme cases, triggering proapoptotic signaling and killing the cell (Wang et al., 2018).

A key player in the ER stress pathway in the heart is the calcium‐binding chaperone calreticulin. Calreticulin is a high‐capacity, low‐affinity calcium‐binding protein localized to the ER lumen in cardiomyocytes (Michalak, Guo, Robertson, Lozak, & Opas, 2004). Calreticulin is upregulated during ER stress in cardiomyoctyes, but the impact of its upregulation is unclear. Overexpression of calreticulin in the heart results in cardiomyopathy, arrhythmia, and heart failure (Hattori et al., 2007; Lee et al., 2013; Mesaeli et al., 2001). The impact of calreticulin seems to be stimulus‐ and cell model‐dependent, and during ischemic heart disease, calreticulin has been identified by different groups as being associated with both cardioprotection and adverse cardiac outcomes under different circumstances (Chen, Wu, Yao, Sun, & Liu, 2011; Cook et al., 2009; Ihara, Urata, Goto, & Kondo, 2006; Karki & Fliegel, 2010; Liu et al., 2013, 2006; Liu, Zhang, Sun, & Wu, 2008; Sun et al., 2017; Wu, Zhang, Jia, & Jia, 2015; Xia et al., 2014; Xu, Liu, & Zhu, 2008; Zhang et al., 2018; Zhao, 2018).

In the studies presented here, we examine the impact of direct modulation of calreticulin in a human‐derived cardiac cell line. We determine that calreticulin upregulation activates apoptotic pathways when ER stress is induced. In addition, we observe that partial knockdown of calreticulin reduces apoptotic ER stress signaling and apoptosis measurements, and reduces total cell death. These data indicate that the reduction of calreticulin expression is protective against ER stress‐driven cardiac injury.

## 2 METHODS

### 2.1 Human tissue

Human heart tissue was obtained from heart failure patients at the University of Pittsburgh Medical Center with informed consent, and with Institutional Review Board approval. Patients had end‐stage chronic ischemic cardiomyopathy (mean age 58 ± 5.8 yrs [*SD*]; 4M/4F), and none were on mechanical support devices. At the time of cardiac transplantation, transmural samples of the lateral wall of the left ventricle (LV) were obtained from failing human hearts (*n* = 8). Nonfailing human heart tissue was collected by the Cleveland Clinic Foundation with Institutional Review Board approval. Transmural samples of the lateral wall of the LV that did not meet the criteria for transplantation were obtained from unmatched donor hearts (*n* = 8) (donor age 51 ± 13.1 yrs [*SD*]; 4M/4F; cause of death, 5 CVA/3 trauma). Cardiac tissue from all groups was placed into cold cardioplegic solution (4°C–8°C) and rapidly transported to the laboratory. Tissues were then snap frozen in liquid nitrogen and stored at −80°C until further analysis.

### 2.2 Cell culture and treatments

AC16 cells were purchased from Millipore. These cells are the product of the fusion of adult human cardiomyocytes with SV40 fibroblasts, producing a cell model that is readily proliferative and retains many of the characteristics of human ventricular myocytes (Davidson et al., 2005). Cells were cultured in DMEM supplemented with 10% FBS as previously described (Manning et al., 2019). To induce ER stress, cells were washed and incubated for 18 hr with the sarco/endoplasmic reticulum Ca2+‐ATPase (SERCA) inhibitor thapsigargin (500nM). For samples intended for further staining, cells were plated onto glass coverslips overnight and then treated as described.

### 2.3 Generation of stable cell lines

Lentiviral transduction particles were purchased from Sigma. To test the effects of calreticulin overexpression, MISSION® TRC3 Human LentiORF particles (Millipore‐Sigma) were used, with ORF‐BFP used as a control. To silence calreticulin expression, MISSION® sh‐RNA lentiviral particles (Millipore‐Sigma) for three different clones of calreticulin were used, and noncoding sh‐RNA was used as a control (sh‐CTR). Cells were incubated with lentiviral particles for 24 hr (MOI = 10), and transduced cells were selected using puromycin (0.5 µg/ml) for 3 weeks. Overexpression or knockdown was subsequently confirmed using reverse transcriptase‐qPCR (RT‐qPCR) and immunoblotting.

### 2.4 Hypoxia and reoxygenation

Hypoxia and reoxygenation (H/R) was conducted as previously described (Manning et al., 2019). In brief, cells were subjected to 1% O_2_, 5% CO_2_, and 94% N_2_ in glucose‐free media containing 2‐deoxyglucose (2‐DG) for 4 hr followed by 2 hr of reoxygenation. All H/R experiments were accompanied by simultaneous normoxia controls for comparison.

### 2.5 Cell survival measurements

To determine cell survival, total cell counts of nontreated/normoxia and treated/HR were quantitated using cell counting kit 8 (CCK8, Millipore‐Sigma) according to the manufacturer's instructions.

### 2.6 Quantitative RT‐PCR

RNA was collected from cells using RNEasy kit (Qiagen) according to the manufacturer's instructions and quantitated using a BioDrop. About 0.5–2 µg of RNA was used to make cDNA using Maxima Reverse Transcriptase (ThermoFisher). Quantitative PCR was then used to determine the expression levels of genes using Qiagen QuantiTect primers for human calreticulin, PPIA, and C/EBP homologous protein (CHOP).

### 2.7 Immunoblotting

Cells were lysed in a 1% CHAPS buffer, and protein was loaded onto an SDS‐PAGE gel. Gels were blotted onto a nitrocellulose membrane, and incubated with primary antibodies overnight Antibodies used were calreticulin (rabbit, 1:1,000, Cell Signaling), α‐tubulin (rabbit, 1:1,000, Cell Signaling), caspase 1 (rabbit, 1:1,000, Cell Signaling), caspase 3 (rabbit, 1:1,000, Cell Signaling), caspase 8 (mouse, 1:1,000, Cell Signaling), caspase 9 (rabbit, 1:1,000, Cell Signaling), P‐PERK (rabbit, 1:1,000, Cell Signaling), PERK (rabbit, 1:1,000, Cell Signaling), XBP1s (rabbit, 1:1,000, Cell Signaling), ATF6 (rabbit, 1:1,000, Cell Signaling), and caspase 12 (rabbit 1:1,000 abcam).

### 2.8 Terminal deoxynucleotidyl transferase dUTP nick end labeling (TUNEL) staining

Treated cells were rinsed with PBS and fixed in 4% paraformaldehyde for 20 min. Cells were permeabilized for 2 min in ice‐cold 0.1% triton. Terminal deoxynucleotidyl transferase dUTP nick end labeling (TUNEL) was performed using the In Situ Cell Death kit –TMR (Roche) according to the manufacturer's instructions, and nuclei were counterstained with DAPI. To quantitate TUNEL‐positive nuclei, 10 random fields were analyzed across three independent experiments per cell line and treatment. TUNEL‐positive nuclei were expressed as a fraction of total DAPI‐stained nuclei for each field.

### 2.9 Statistical analysis

All data were analyzed using GraphPad Prism. Student's *t* tests were used to compare two data sets, and Analysis of Variance (ANOVA) tests followed by post hoc Dunnett's multiple comparison tests or student's *t* tests were used to compare two or more experimental groups to a control group. A p value of less than 0.05 was taken as significant.

## 3 RESULTS

### 3.1 Ischemic heart disease, hypoxia/reoxygenation, and thapsigargin induce calreticulin expression

**Figure 1:**
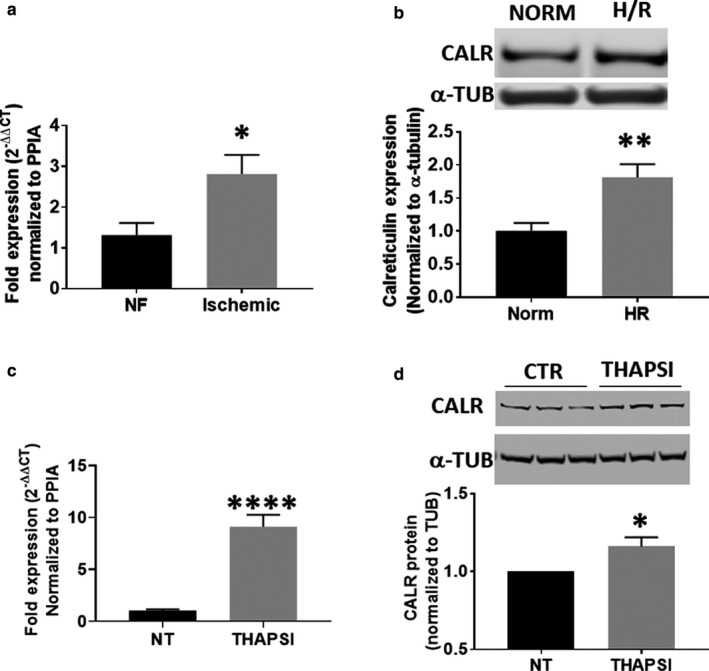
(a) Reverse transcriptase‐quantitative PCR (RT‐qPCR) analysis of human tissue reveals a significant increase in calreticulin mRNA expression in the hearts of patients with ischemic heart failure over nonfailing. (b) Immunoblotting shows that acute hypoxia and reoxygenation (H/R) drives the induction of calreticulin protein in AC16 cells. (c) Treatment of AC16 cells with thapsigargin (500 nM, THAPSI) drives an increase in calreticulin mRNA as measured by RT‐qPCR, compared to no treatment controls (NT). (d) Immunoblots showing an increase in calreticulin protein levels in the presence of thapsigargin. *p < .05, **p < .01, ****p < .0001 compared to control

Calreticulin has been previously reported to be upregulated in response to ischemia‐reperfusion (IR) injury. To identify whether this increase is found in human tissue, we examined samples acquired from patients with ischemic heart disease, and measured calreticulin mRNA. We found that calreticulin expression was upregulated in these hearts (Figure 1a), establishing a foundation of clinical relevance for further studies examining the impact of calreticulin modulation on survival. We next examined whether acute hypoxia/reoxygenation studies are sufficient to cause a significant increase in calreticulin. We used AC16 cells, a human‐derived proliferating cardiomyocyte line, subjected to glucose and oxygen deprivation followed by reoxygenation. We found that 4 hr is sufficient to promote an increase in calreticulin protein expression (Figure 1b). We were next able to mimic this calreticulin upregulation by inducing ER stress directly using the sarco/endoplasmic reticulum Ca2+‐ATPase (SERCA) inhibitor thapsigargin (Figure 1c,d). These data suggest that calreticulin upregulation is a common feature of ischemic and ER stress‐driven cardiac injury.

### 3.2 Overexpression of calreticulin drives proapoptotic signaling in the presence of ER stress in cardiac cells without protecting from cell death

**Figure 2:**
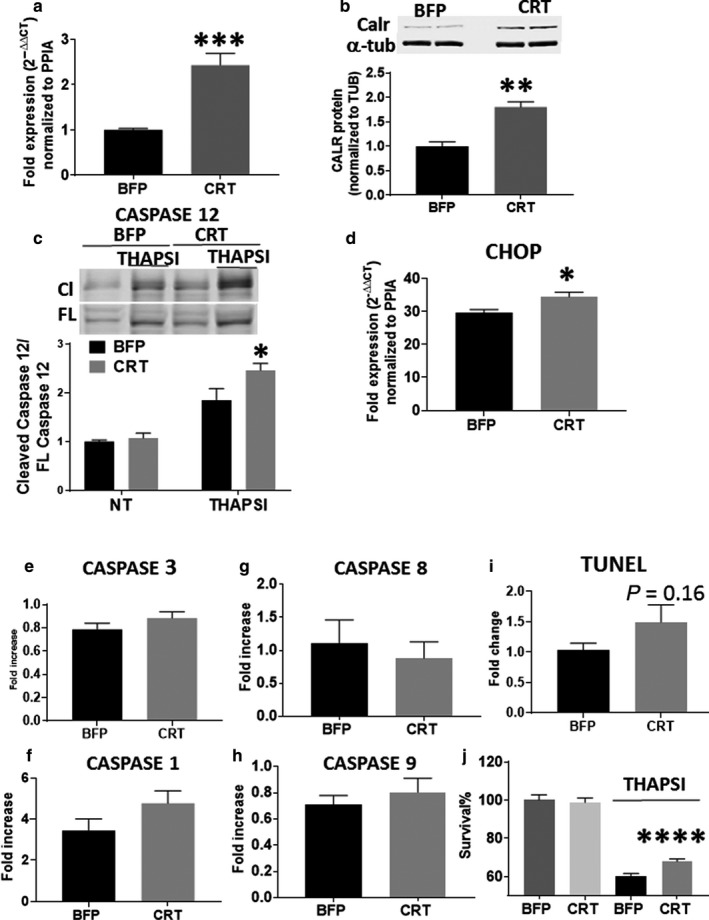
(a and b) RT‐qPCR analysis (a) and immunoblotting (b) confirm stable overexpression of calreticulin in AC16 cells (CRT) compared to control transduced cells (BFP). (c) Caspase cleavage as measured by immunoblot is significantly increased in CRT cells compared to BFP cells in the presence of thapsigargin. Cl: cleaved caspase 12, FL: full length caspase 12. (d) CHOP mRNA measured by RT‐qPCR is increased in CRT cells compared to BFP controls. (e–h) No difference in caspase 3 (e), caspase 1 (f), caspase 8 (g), or caspase 9 (h) expression was observed between CRT and BFP cells exposed to thapsigargin. (i) No significant difference in TUNEL staining was observed between CRT and BFP exposed to thapsigargin. (j) The survival of CRT cells exposed to thapsigargin is significantly higher than BFP controls. *p < .05, **p < .01, ***p < .001, ****p < .0001 compared to BFP cohorts

AC16 cells were transduced with lentiviral particles to stably overexpress calreticulin (Figure 2a,b). ER stress was induced via thapsigargin incubation for 18 hr. We found that thapsigargin treatment increased proapoptotic CHOP expression and CRT overexpression further increased/enhanced the expression of CHOP (Figure 2d). Similarly, overexpression of calreticulin was associated with increased caspase 12 cleavage (Figure 2c), although no change was found in the induction of caspase 1, caspase 3, caspase 8, or caspase 9 (Figure 2e‐h). Although a ~50% increase in cell death in calreticulin overexpressing cells was observed using TUNEL staining, this trend did not reach statistical significance (*p* = .16; Figure 2i). However, we found that despite the activation of apoptotic signaling events, calreticulin overexpression results in increased survival of cells subjected to thapsigargin treatment (Figure 2j). Overall, these data suggest that apoptosis signaling in response to ER stress is upregulated when calreticulin is overexpressed, but that exogenous overexpression of calreticulin also protects against cell death.

### 3.3 Partial knockdown of calreticulin protects against ER stress‐induced apoptosis in cardiac cells

**Figure 3:**
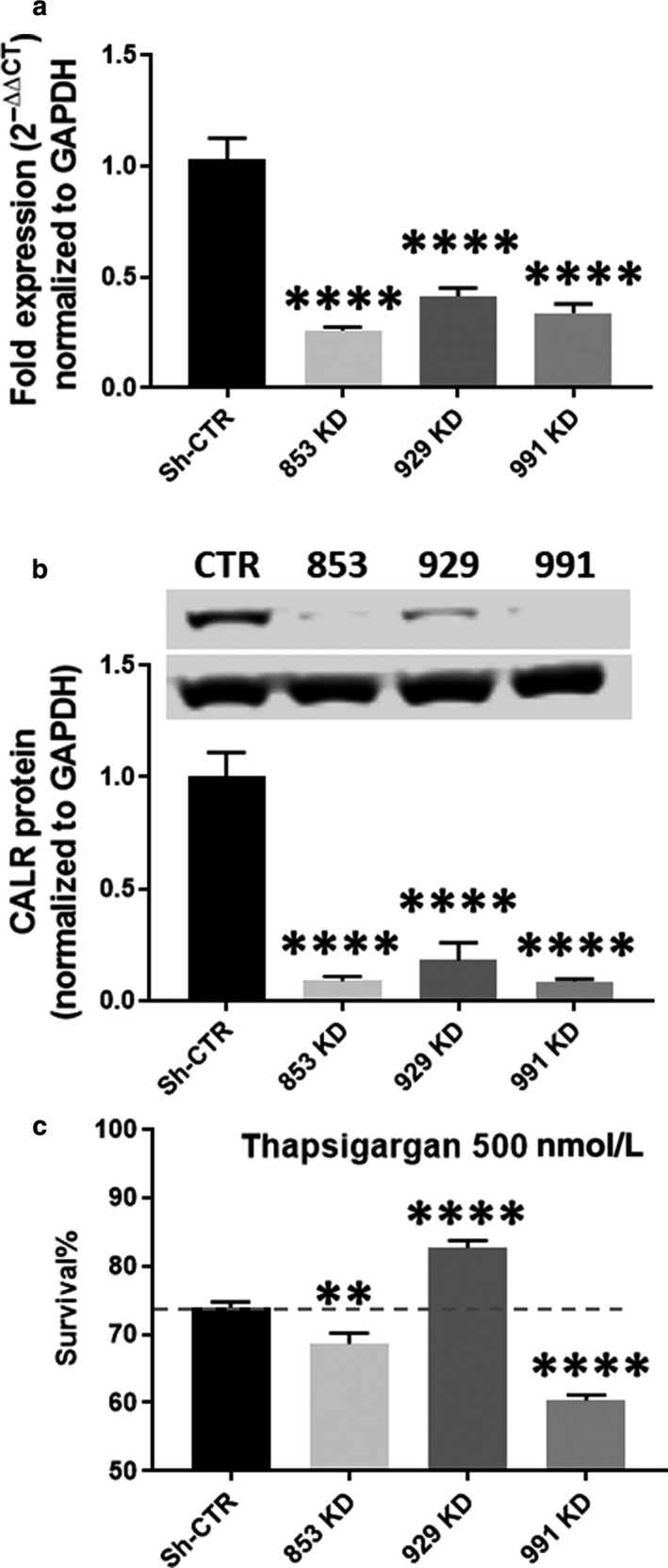
(a) RT‐qPCR analysis of calreticulin mRNA in control (SH‐CTR) and three knockdown lines of AC16 cells (853 KD, 929 KD, and 991 KD). (b) Immunoblotting reveals varying levels of calreticulin expression in knockdown cell lines. (c) In cells subjected to thapsigargin treatment, cell survival is increased in the 929 KD line, but decreased in lines 991 KD and 853 KD, respectively. **p < .01, ****p < .0001 compared to SH‐CTR cohorts

**Figure 4:**
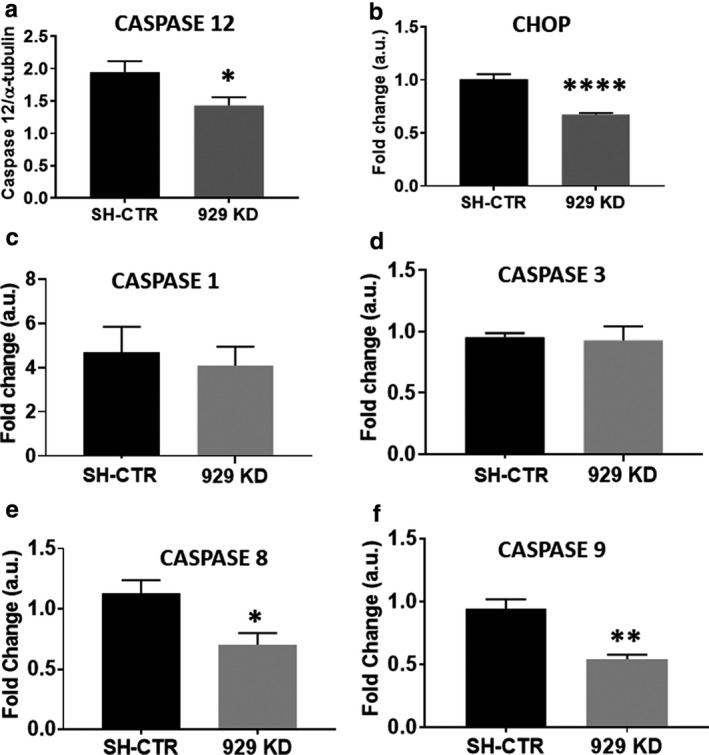
(a) Caspase 12 induction by thapsigargin treatment as measured by immunoblot is decreased in the 929 KD partial calreticulin knockdown cell line. (b) CHOP mRNA induction is similarly decreased in the 929 KD partial knockdown line. (c and d) Caspase 1 (c) and caspase 3 (d) induction measured by immunoblot is similar in both lines. (e and f) Cleaved caspase 8 (e) and caspase 9 (f) are significantly reduced compared to control cells after thapsigargin treatment. *p < .05, **p < .01 ****p < .0001, compared to SH‐CTR cohorts

**Figure 5:**
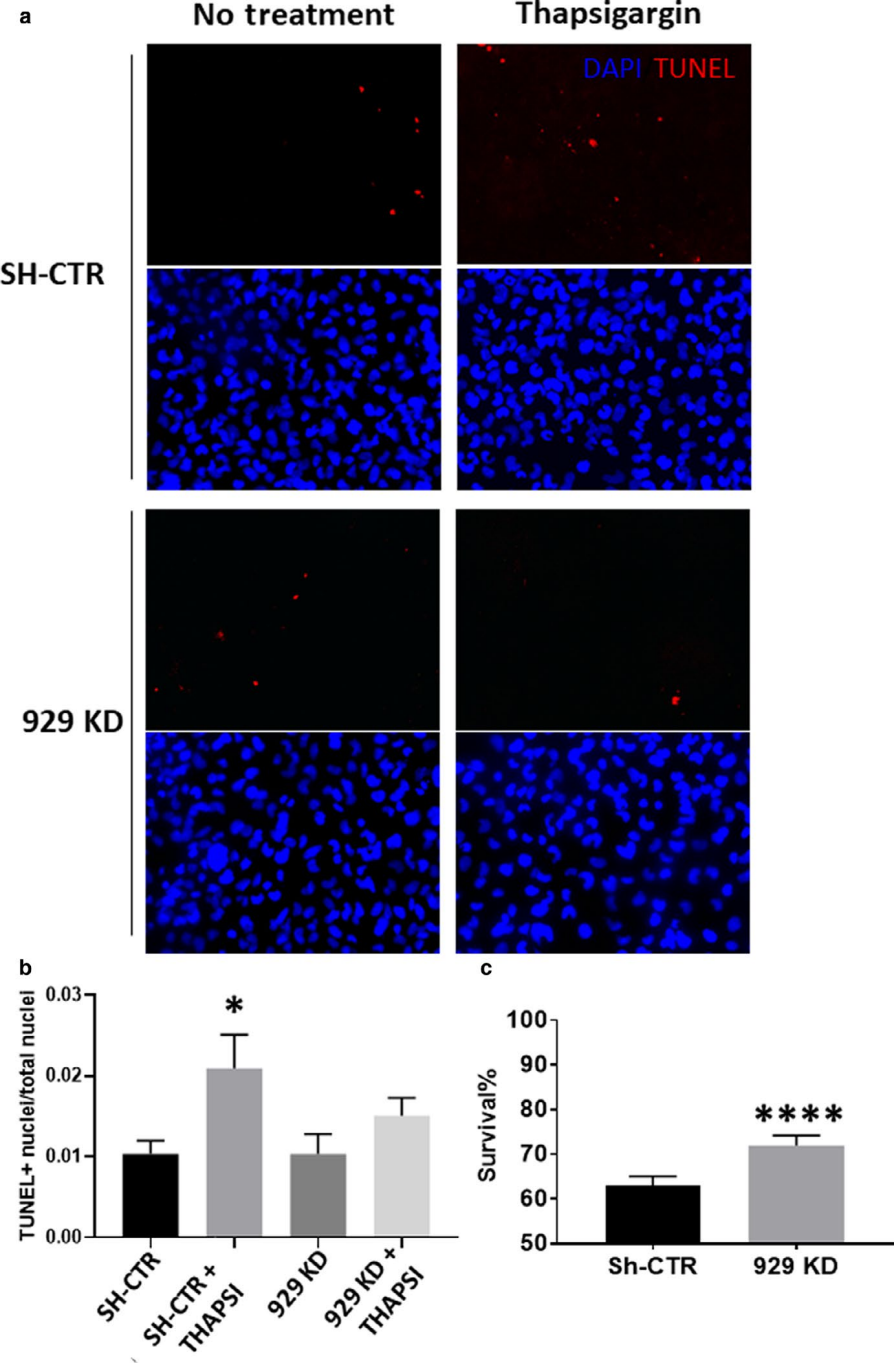
(a) Representative images of cells subjected to no treatment (left column) or thapsigargin (right column) stained with TUNEL (red) or DAPI (blue). (b) TUNEL counts show that sh‐CTR cells exhibit significantly more TUNEL‐positive nuclei when exposed to thapsigargin, while, 929 KD cells do not. (c) Survival is significantly increased in 929 KD cells after ER stress. *p < .05 ****p < .0001 versus controls

We next switched to a loss‐of‐function model to determine whether silencing calreticulin attenuates proapoptotic signaling. We generated three lines of AC16 cells transduced with sh‐RNA to produce varying levels of calreticulin reduction (Figure 3a,b). Cell line 929 KD expressed approximately 17% calreticulin protein relative to the control, while both 853 KD and 991 KD expressed approximately 8% (Figure 3a,b). Interestingly, we observed a decrease in the survival of two of our cell lines expressing nearly undetectable levels of calreticulin, while observing an increase in the survival of the partial 929 KD line (Figure 3c). We hypothesized that while some calreticulin is necessary for cell viability, a partial knockdown would reduce apoptotic signal induction. To test this, we measured apoptotic markers in our partial knockdown. We found that partial knockdown of calreticulin reduced CHOP and caspase 12 induction (Figure 4a,b). No change was observed in the induction or cleavage of caspases 1 and 3 (Figure 4c,d). As caspases 8 and 9 are both downstream of apoptotic signaling induced by ER stress, we next examined the impact of partial calreticulin knockdown on these caspases. We found that with thapsigargin treatment, caspase 8 and caspase 9 were both reduced, suggesting that these may lie downstream of caspase 12 (Figure 4e,f). To determine the effect of calreticulin silencing on apoptosis, we evaluated cells subjected to ER stress using TUNEL staining. We found that the number of TUNEL‐positive nuclei was reduced in our partial knockdown, confirming that apoptosis is decreased by partial calreticulin knockdown in AC16 cells (Figure 5a,b), and this decrease occurred in association with increased survival (Figure 5c).

**Figure 6:**
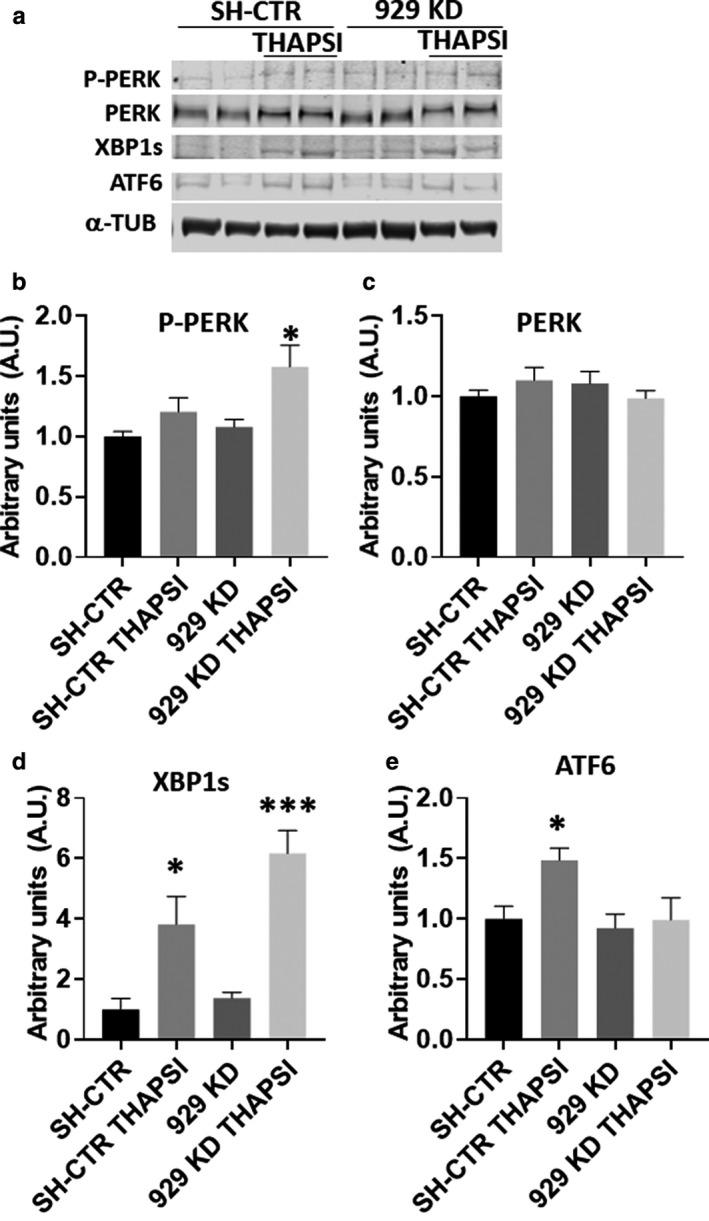
(a) Representative immunoblots of ER stress proteins. (b) P‐PERK is significantly increased in 929 KD lines after thapsigargin. (c) No change is observed in total PERK. (d) XBP1s is significantly increased after thapsigargin in both knockdown and control lines. (e) ATF6 is significantly increased in the control cells, but not in the knockdown lines. *p < .05, ***p < .001 versus no treatment controls

We next sought to determine the impact of partial calreticulin knockdown on the activation of each branch of the ER stress pathway (Figure 6a). A significant increase in the phosphorylation of PERK was observed in the knockdown after thapsigargin (Figure 6b), with no significant difference in total PERK (Figure 6c). No difference was observed in XBP1s production, indicating no change in IRE‐1 pathways (Figure 6c). However, ATF‐6 detection was significantly increased after thapsigargin in control cells, while no increase was observed in the knockdown cells (Figure 6d).

## 4 DISCUSSION

In this manuscript, we directly test for the first time the effects of modulating calreticulin expression levels on the survival of human cardiac cells in response to ER stress. We demonstrate that calreticulin upregulation in the context of ischemic heart disease, in vitro hypoxia, and direct induction of ER stress may play an important role in the activation of apoptotic cell death pathways. Directly increasing calreticulin expression under ER stress‐inducing stimuli activates proapoptotic pathways, without resulting in more cell death. Conversely, partial reduction of calreticulin reduces apoptotic signaling, apoptosis, and overall cell death in response to ER stress, although nearly complete knockdown of calreticulin fails to protect cells in a similar fashion. In addition, changes in upstream ER stress proteins PERK and ATF6 are observed. These data shed light on the contradictory effects that have been attributed to calreticulin under conditions of cardiac ischemia.

Establishing a firm causative link between calreticulin expression and either pro‐ or anti‐survival mechanisms in cardiomyocytes exposed to ischemia or ER stress has proven elusive. Examining the association between protective or injurious stimuli and calreticulin expression has yielded mixed results. Supporting a protective role for calreticulin is the report that adiponectin simultaneously increases calreticulin expression and reduces apoptosis (Sun et al., 2017). Similarly, CTRP9 has been reported to activate PKA and block apoptosis via association with calreticulin (Zhao, 2018). In contrast, hypercholesterolemia in conjunction with IR increases both calreticulin levels and apoptosis (Wu et al., 2015) Similarly, atorvastatin protects the heart from IR injury and downregulates ER stress proteins, including calreticulin (Xia et al., 2014). *Panax quinquefolium* extract reduces both calreticulin expression and apoptotic signaling after ischemia (Liu et al., 2013). In a model of canine microembolism of the left anterior descending coronary artery, the resultant heart failure was associated with increased calreticulin and other markers of injurious ER stress including eIF2a phosphorylation (George, Sabbah, Xu, Wang, & Wang, 2011). Treatment with metoprolol rescued cardiac function, and simultaneously reduced calreticulin levels (George et al., 2011). Similarly, multiple groups have found that IR injury in models of NHE1 overexpression results in increased ER stress protein expression, including calreticulin. Cook and colleagues found a cardioprotective phenotype (Cook et al., 2009), while Karki and colleagues found that calreticulin upregulation was associated with increased apoptosis (Karki & Fliegel, 2010).

In addition to pharmacological and transgenic approaches, pre‐ and postconditioning are powerful tools to protect the heart from ischemic injury, but the reported role of calreticulin expression in these interventions is contradictory as well. Hypoxic preconditioning in rat neonatal cardiomyocytes has been reported to upregulate calreticulin via p38, and blockade of p38 reverses hypoxic preconditioning (Liu et al., 2006; Wu, Liu, Zhu, & Tang, 2007). Conversely, both Chen and colleagues and Liu and colleagues have shown that ischemic postconditioning reduces calreticulin expression in the myocardium (Chen et al., 2011; Liu, Zhang, et al., 2008).

Experiments directly modulating calreticulin expression are in theory a more direct way to show a causative association between calreticulin and survival from ischemia and ER stress, but these experiments have been similarly contradictory. Overexpression of calreticulin in H9c2 cells exacerbates simulated ischemia via H_2_O_2_‐induced oxidative stress, (Ihara et al., 2006) while in contrast, the protection afforded by hypoxic preconditioning from H_2_O_2_ is abrogated by silencing calreticulin in rat neonatal myocytes (Xu et al., 2008).

Our experiments attempt to resolve this paradox by directly altering calreticulin expression in both a loss‐of‐function and a gain‐of‐function model. The finding that increasing calreticulin expression activates proapoptotic caspase 12 and CHOP indicates that calreticulin induction in response to ER stress increase signaling cascades that typically drive programmed cell death. However, increased survival was observed when calreticulin was overexpressed, and no change in TUNEL staining was observed. These data point to an uncoupling of apoptotic signaling from overall survival. Although unexpected, increased apoptosis signaling failing to correspond to higher cell mortality has been reported in the literature. For example, although hypoxic insult results in cytochrome C release from glioma cells, apoptosis inhibition using X‐linked inhibitor of apoptosis protein (XIAP) or caspase inhibitors fails to protect cells from hypoxic cell death (Steinbach, Wolburg, Klumpp, Probst, & Weller, 2003). The presence of apoptotic markers without increased cell death suggests that exogenous calreticulin overexpression in AC16 cells does not produce a straightforward antiapoptotic effect, and the mechanism for this paradox remains unclear.

In addition, a moderate decrease in calreticulin expression was found both to reduce overall cell death and reduce apoptotic markers. This suggests that the endogenous expression of calreticulin in response to ER stress is cytotoxic overall, and blockade of endogenous expression is protective. These results stand in contrast to the overexpression model, where survival is uncoupled from apoptotic signaling. The protective effects of calreticulin knockdown are further supported by the finding that pro‐death caspases 8 and 9 are reduced in the knockdown model, while these were not found to be changed in the overexpressing cells. However, some amount of calreticulin seems to be necessary for survival, as a nearly complete knockdown of calreticulin resulted in more cytotoxicity and overall reduced viability. Rather, only a modest decrease in the amount of calreticulin expression results in improved survival.

To evaluate the role of apoptosis in our partial knockdown, a multifaceted approach was used, making use of both TUNEL and the activation of proapoptotic pathways (Mishra et al., 2019). Differences were found in both the activation of CHOP and caspase 12 when calreticulin expression is altered. CHOP is induced by ATF4 downstream of the PERK/eIF2a cascade, and regulates the expression of Bcl‐2 proteins, driving mitochondrial apoptosis (Li, Guo, Tang, Jiang, & Chen, 2014). Importantly, CHOP deletion has been linked to reduced infarct development after ischemia, indicating that it is a key mediator of ischemic injury (Miyazaki et al., 2011). Caspase 12 is a caspase protein family member specifically associated with ER stress stimuli (Ferri & Kroemer, 2001). Like other caspases, caspase 12 is induced and cleaved in response to apoptotic stimuli. Caspase 12 inhibition has also been associated with improved outcomes in heart failure (Liu et al., 2014; Zhao et al., 2013). While thapsigargin was also found to induce caspase 1 in all cell lines, we found no effect of modulating calreticulin expression on its induction. In addition, we were unable to detect differences in caspase 3 induction or cleavage. These data suggest that calreticulin is specifically driving ER stress‐initiated apoptotic signaling. TUNEL allows the visual identification of double‐strand DNA breaks characteristic of apoptosis, and is useful as a measurement of late‐stage apoptotic cell death regardless of which signaling pathways are activated. The discovery that partial knockdown of calreticulin reduces these apoptotic markers, as well as overall cell survival, indicates an overall cytoprotective effect of reducing calreticulin induction in response to ER stress. The direct mechanisms by which calreticulin activates the proapoptotic signaling identified in this manuscript remain the subject of further study.

In addition to acting as a chaperone for nascent proteins, calreticulin acts as a low‐affinity, high‐capacity calcium‐binding molecule in the ER. As a result, manipulating calreticulin levels results in changes in calcium levels in the ER and cytosol. Loss of calreticulin results in decreased luminal and cytosolic calcium measurements (Faustino et al., 2016; Nakamura et al., 2001). Calcium plays a key role in the induction of ER, as sufficient calcium levels are required for proper protein folding and ER calcium depletion. The mechanism of inducing ER stress in this manuscript is to block the sarco/endoplasmic reticulum ATPase (SERCA), and thapsigargin depletes cultured cardiomyocytes of ER calcium within 10 min (Zhao, Li, Brochet, Rosenberg, & Lederer, 2015). Interestingly, reduction of calreticulin may be expected to accelerate intraluminal calcium loss and exacerbate injury. Instead, we observe the opposite, where apoptosis driven by SR calcium depletion‐induced ER stress is mitigated by reduced calreticulin. This suggests that calcium buffering alone is not the cause of calreticulin's effects on the survival of the heart cell, and points to specific pathways being activated as ER stress is induced.

In probing the direct response of the ER stress pathway to calreticulin knockdown, changes were observed in two of the three arms. No change was observed in spliced XBP1, suggesting that no change was observed in the IRE‐1 pathway. An increase in PERK phosphorylation was observed. Although PERK is often associated with increased apoptosis and CHOP induction in heart cells (Prola et al., 2017; Sun et al., 2019; Wang, Jia, Zhang, & Wang, 2017), noncanonical signaling may result in the secondary upregulation of protective factors, such as VEGF (Liu et al., 2019) In addition, ATF6 induction was abrogated after thapsigargin treatment, suggesting that calreticulin plays a role in the stimulation of this pathway during ER stress. ATF6 activation has been reported to mediate ER stress‐induced injury in several models, indicating that this may be a likely candidate for linking calreticulin to apoptosis (Correll et al., 2019; Sun et al., 2019; Zhou et al., 2020). Further study is required to determine whether PERK and ATF6 play a direct causal role in the cytoprotection induced by partial calreticulin knockdown.

In addition to ischemic heart disease, the results described here may also be relevant for other cardiac diseases as well. ER stress and calreticulin have been described as important players in heart failure associated with numerous etiologies. For example, left ventricular assist device unloading in dilated cardiomyopathy reduces cardiac stress and reduces ER stress‐induced injury markers, which correlate to improved LV reverse remodeling (Castillero et al., 2015). A rat model of posttraumatic stress disorder results in increased calreticulin expression and increased ER‐related apoptotic markers, likely through a catecholaminergic mechanism (Liu et al., 2016). Calreticulin upregulation has also been linked to calcineurin activation in cardiomyocytes, which mediates hypertrophic heart failure (Chen et al., 2011; Guo et al., 2002; Liu, Wu, Cai, & Sun, 2008; Lynch, Chilibeck, Qui, & Michalak, 2006; Lynch et al., 2005; Lynch & Michalak, 2003; Mesaeli et al., 1999; Zhang, Liu, Hu, Rong, & Wu, 2010). The discovery that calreticulin induces apoptotic signaling while simultaneously protecting cardiac cells from cell death when overexpressed or severely downregulated may shed light on the mechanisms driving these and other cardiac diseases as well.

## CONFLICT OF INTEREST

The authors report nothing to disclose.

## Supporting information



Fig S1‐S3
