# Supplementary material for: Calreticulin expression in human cardiac myocytes induces ER stress‐associated apoptosis
Source: Physiol Rep. 2020 Apr 23;8(8):e14400. doi: 10.14814/phy2.14400 (PMC7177173; doi:10.14814/phy2.14400)

Figure 1B:

Calreticulin

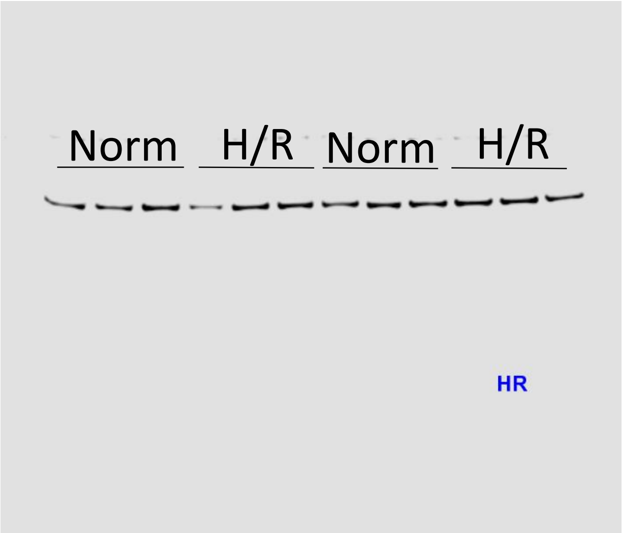

Tubulin

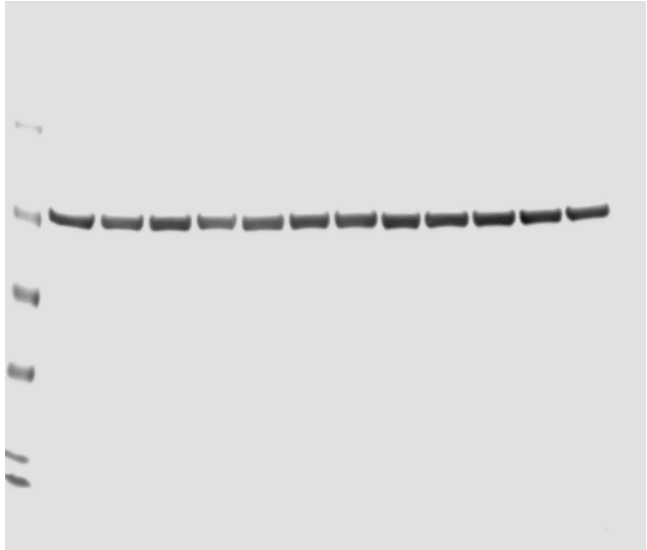

Figure 1D:

Calreticulin

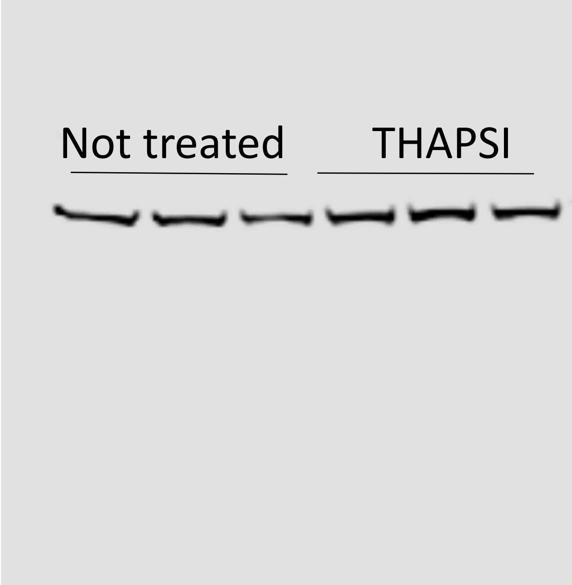

Tubulin

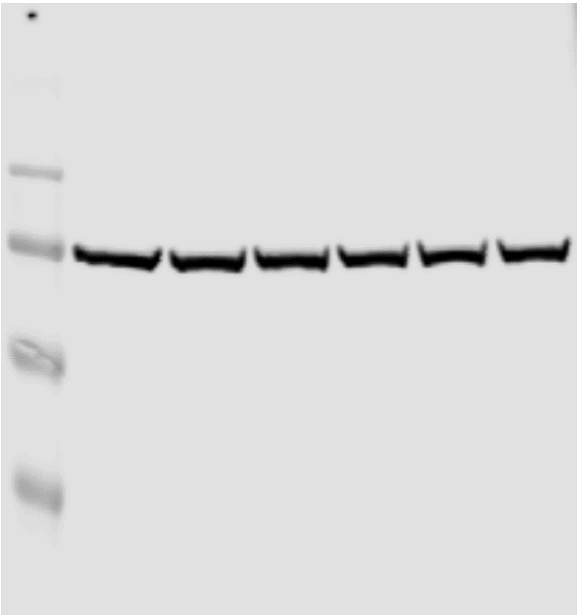

Figure 2B

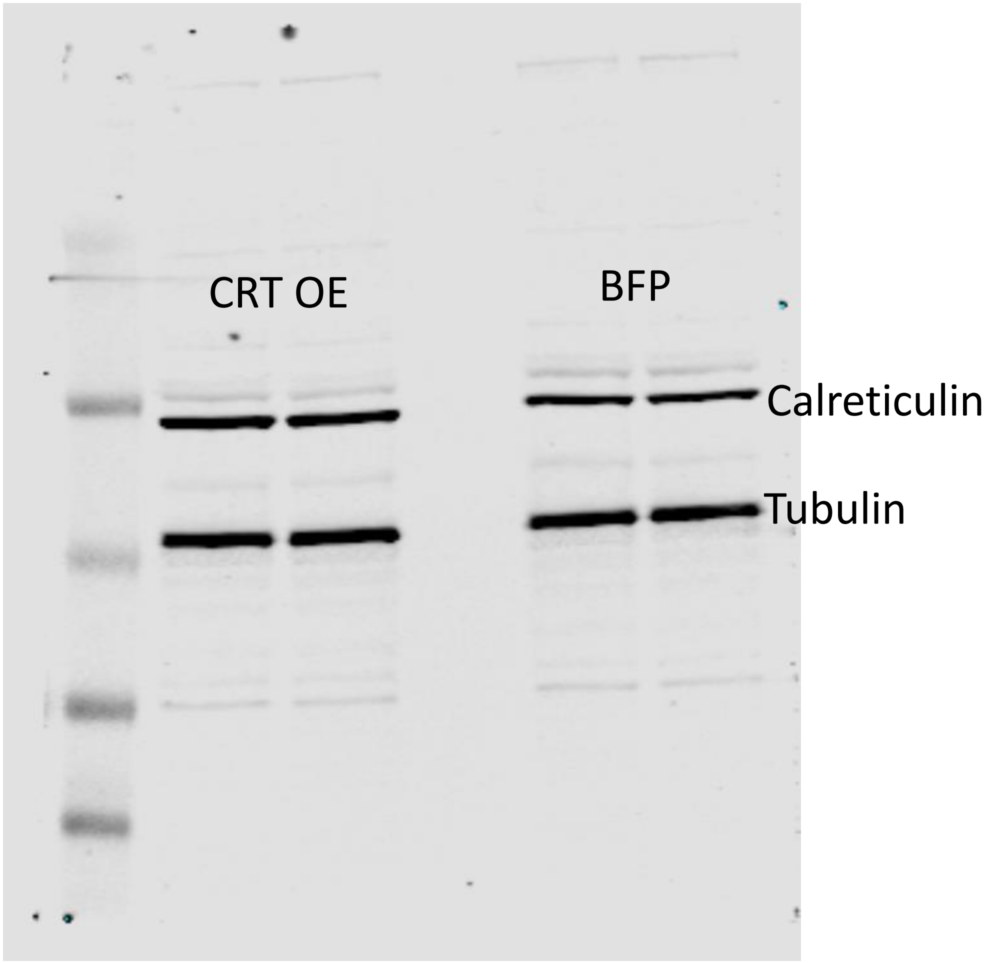

Figure 2C

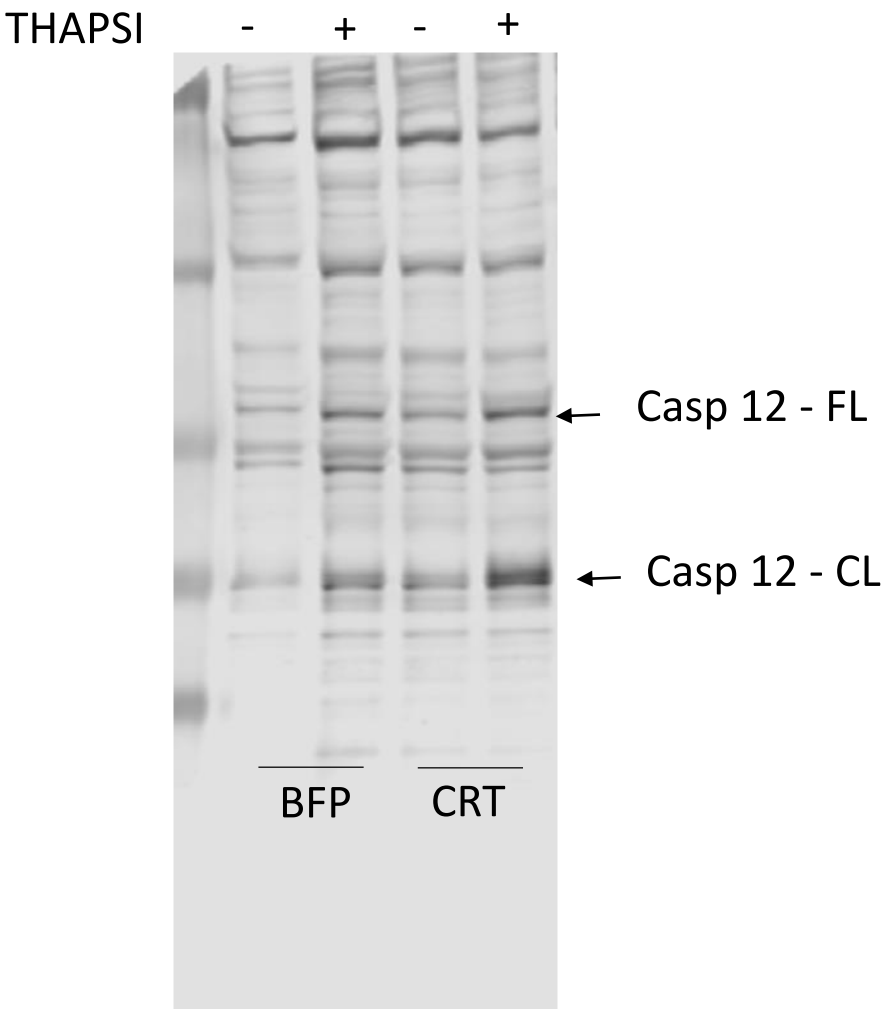

Figure 3B

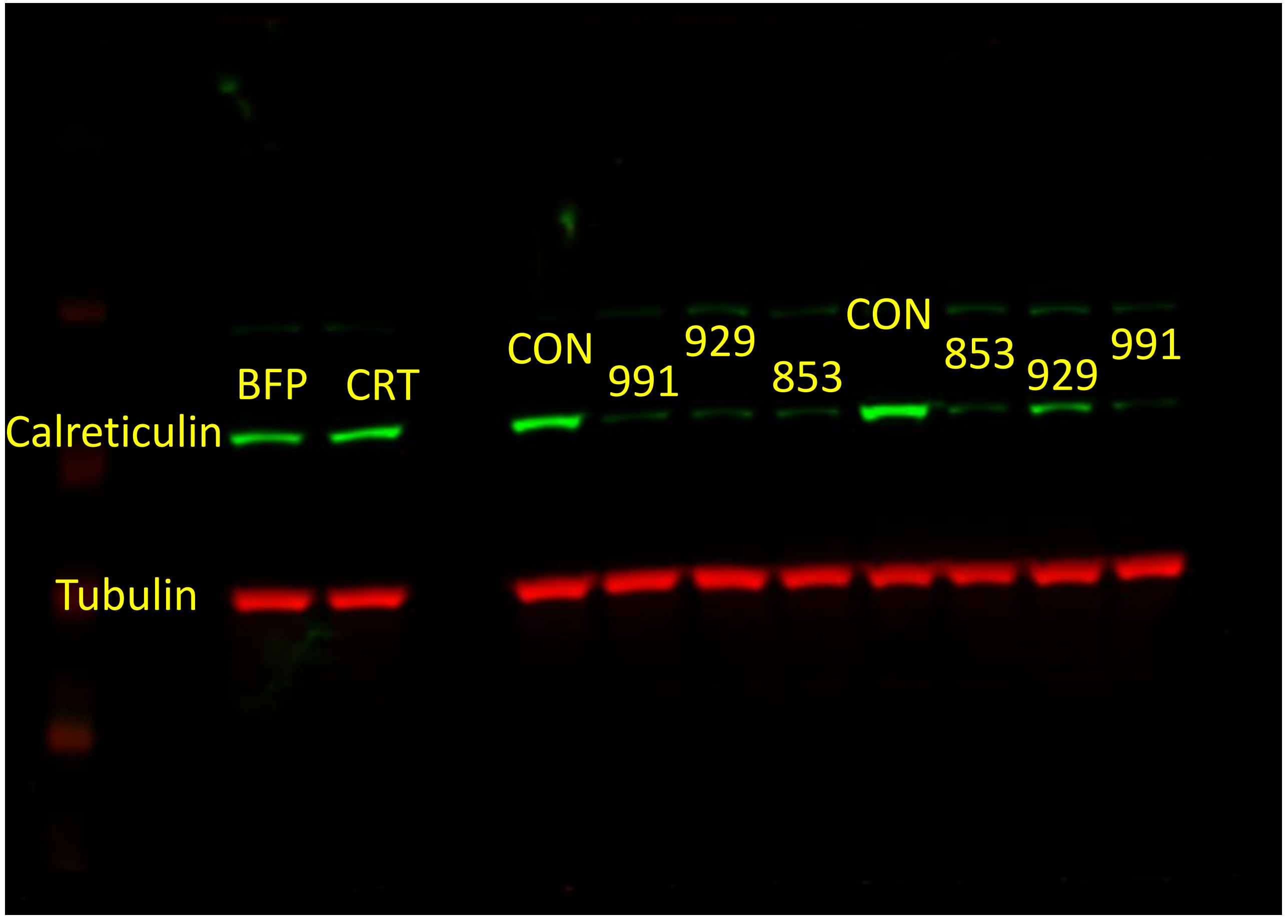

Figure 5A

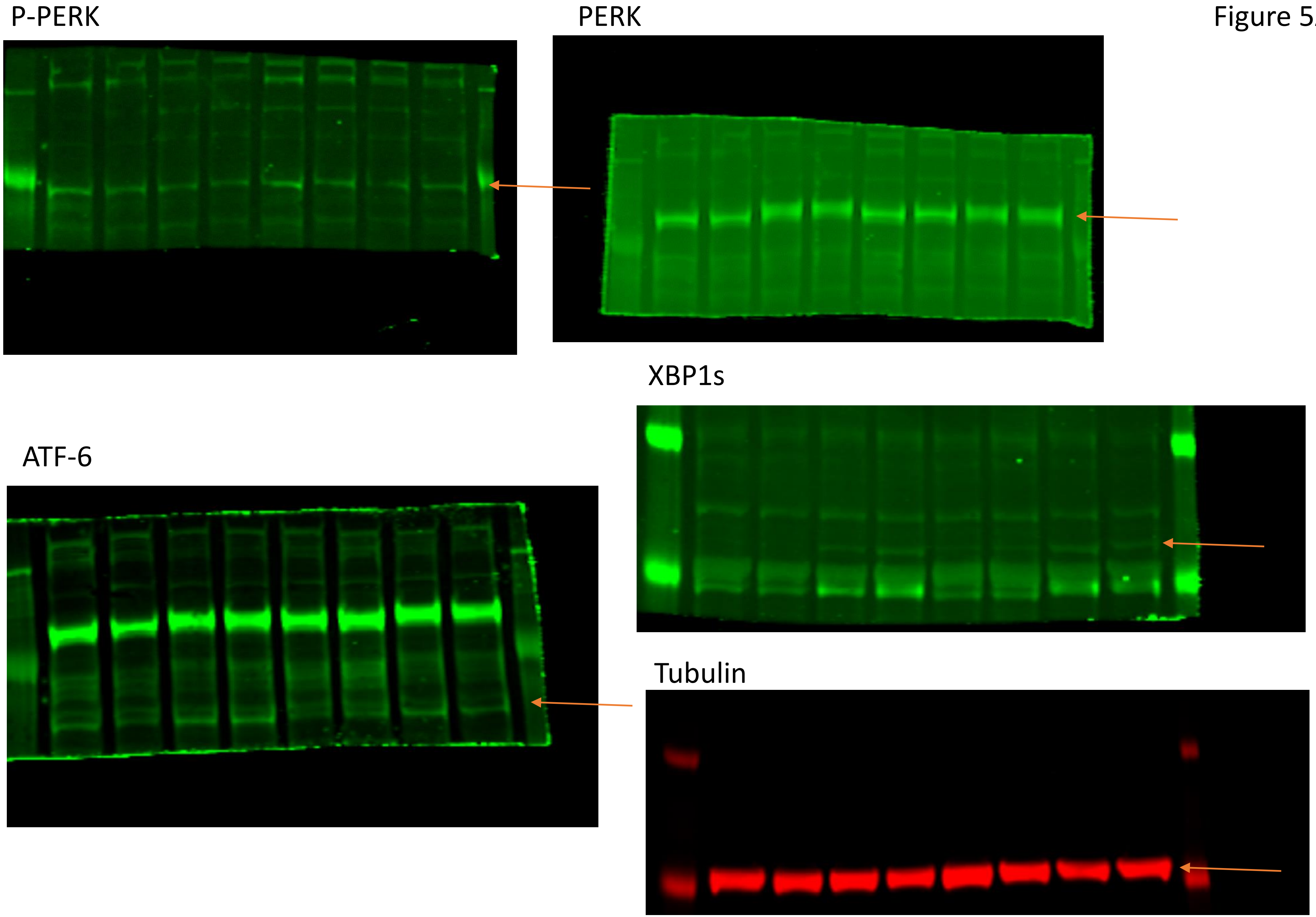

Supplement: Supplementary file 1 — Fig S1‐S3 [file PHY2-8-e14400-s001.pdf]
